# Carbon nanotube impregnated anthracite (An/CNT) as a superior sorbent for azo dye removal

**DOI:** 10.1039/d0ra03869e

**Published:** 2020-07-07

**Authors:** Fathy M. Mohamed, Zhaohui Li, Ahmed M. Zayed

**Affiliations:** Water and Environment Department, Faculty of Earth Sciences, Beni-Suef University Egypt; Geosciences Department, University of Wisconsin – Parkside Kenosha WI 53144 USA; Applied Mineralogy and Water Research Lab, Department of Geology, Faculty of Science, Beni-Suef University Egypt zayed_2000eg@yahoo.com ahmed.zayed@science.bsu.edu.eg

## Abstract

Raw anthracite was impregnated with a minute amount of multi-walled carbon-nanotubes at a solid/solid ratio of 50 : 1 *via* calcination at 950 °C for 2 h to produce anthracite/carbon nanotube (An/CNT) composite with superior sorption efficiency. Both An/CNT composite and its precursor anthracite were characterized by XRD, SEM, FT-IR and BET surface area (*S*_BET_). The removal efficiency of an azo dye methyl orange (MO) by the An/CNT composite was evaluated under different experimental parameters. The MO sorption isotherm data fitted to the Langmuir model well with an *R*^2^ of 0.999 and a MO sorption capacity (*q*_max_) of 416.7 mg g^−1^. The distribution coefficient *K*_d_ decreases from 117.9 to 16.1 L g^−1^ as the initial MO concentrations increased from 40 to 140 mg L^−1^. The MO sorption kinetic data was well described by the pseudo-second-order equation with an *R*^2^ of 1. The external (film) diffusion followed by intra-particle diffusion was the major driving process during the early stage of MO sorption. The electrostatic interaction between the oxygen- and nitrogen-bearing functional groups on the An/CNT surface and MO ions was the key controlling mechanism for the MO sorption process, particularly at pH < pH_PZC_ of the composite. Meanwhile, valuable contributions from Yoshida and dipole–dipole H bonding mechanisms can explain the MO sorption by the addressed composite, especially at pH > pH_PZC_.

## Abstract 2

Raw anthracite was impregnated with a minute amount of multi-walled carbon-nanotubes at a solid/solid ratio of 50 : 1 *via* calcination at 950 °C for 2 h to produce anthracite/carbon nanotube (An/CNT) composite with superior sorption efficiency.

## Graphical abstract

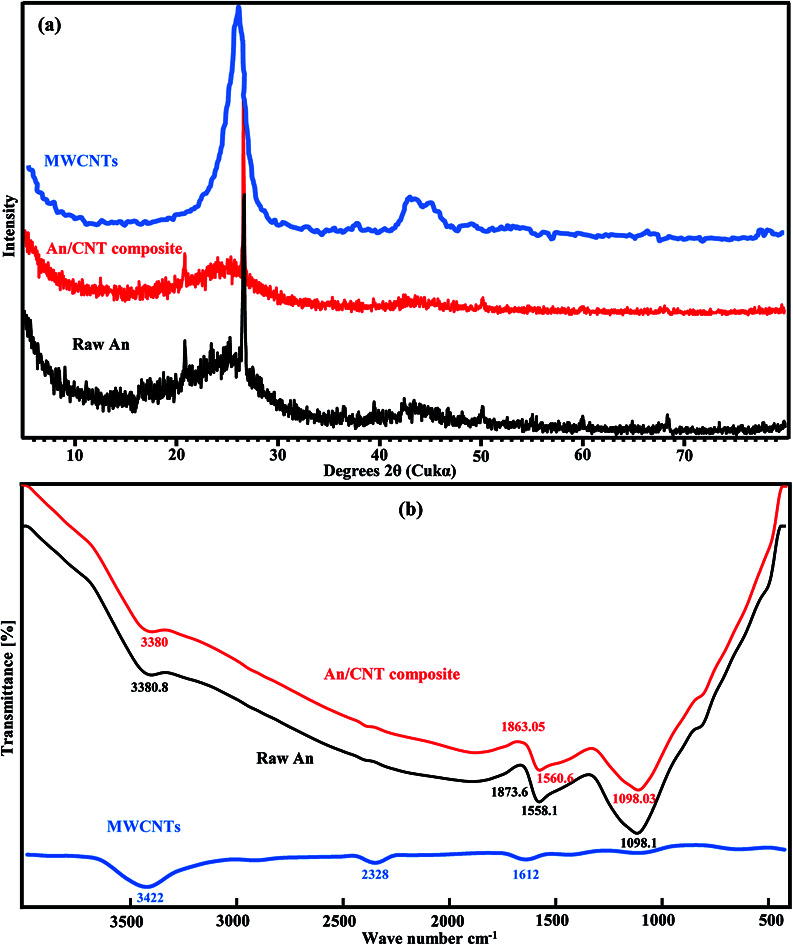

## 1. Introduction

Methyl orange (MO) is extensively used in several industries such as paper, rubber, cosmetic, plastic, leather, pharmacy and food industry.^1^ The MO contaminated wastewater effluent from these industries has become a serious environmental challenge due to its carcinogenic and mutagenic effects on human health.^2,3^ Consequently, MO removal from the effluent before discharge into the aquatic environment requires effective and revolutionary solutions.^4,5^

In spite of the success of MO remediation at the laboratory scale based on photo-catalysis,^4^ electrochemical oxidation^6^ and ultrafiltration techniques,^7^ at the industrial scale MO removal did not achieve the same success due to some economical and technical reasons. On the other hand, sorption techniques showed very good applicability in MO removal with high efficiency and reasonable costs.^8^

Carbon nanotubes (either single or multi-walled) are well-known efficient sorbents for the remediation of both inorganic and organic wastewater's contaminants. As such, numerous studies were devoted to extend their uses for the removal of color dyes form aqueous solution.^9–11^ The sorption of MO from aqueous solutions onto functionalized multi-walled carbon nanotubes (MWCNTs) was investigated as a function of contact time, dosage, temperature, pH, and MO concentrations in the solution with an MO sorption capacity of 53 and 67 mg g^−1^.^8,12^ Uptake of MO on MWCNTs was very fast with a pseudo first order rate constant of 0.02–0.03 min^−1^.^13^ Their efficiency was attributed to their large specific surface area (SSA) and meso-porosity.^8,14^ However, carbon nanotubes are expensive to fabricate. Anthracite (either raw or activated) is an inexpensive natural raw material of low cost. Its use as filtration media can be dated back to 1933.^15^ However, its application for contaminant removal from water has not been well explored. Moreover, its removal of an azo dye Acid Yellow 42 showed a capacity of on 9 mg g^−1^.^16^ In this study, anthracite (An) was impregnated with multi-walled carbon nano-tubes (MWCNTs) first at a solid/solid ratio of 50/1. The An/CNT composite was tested for the MO removal from aqueous media under different physico-chemical conditions to: (1) evaluate its MO removal efficiency, (2) highlight the role of the utilized MWCNTs in improving the removal efficiency of MO by the prepared composite, (3) assess the impact of the applied experimental parameters (pH, adsorbent dose, initial MO concentration, agitation time and speed) on MO sorption by An/CNT composite, (4) elucidate the mechanism and the behavior of MO sorption by fitting the experimental outputs to some well-known kinetic and isotherm models, and finally (5) assess the impact of the sorbed MO on the morphological characteristics of the sorbent An/CNT composite.

## 2. Materials and methods

### 2.1. Materials

Raw anthracite (An) was obtained from Matrouh Company, Matrouh Governorate, Egypt. MWCNTs with carbon content >97% and metal basis traces <3% were purchased from the Science and Technology Center of Excellence, National Organization for Military Production, Egypt. Although functionalizing MWCNTs will increase their versatility and efficiency, it will inevitably increase the overall material cost. As such, the MWCNTs were used as is without functionalization. MO was purchased from Fluka (Switzerland). It has a molecular formula of C_14_H_14_N_3_SO_3_Na with a formula mass of 327.33 g mol^−1^. Other chemicals used in the experiments include 0.01 M HCl and 97% NaOH (Alfa Aesar) for pH adjustment.

### 2.2. Preparation of An/CNT composite

Bulk raw An (2 kg with a particle size of 2–3 mm) was quartered several times to homogenize the sample. The selected quarter (100 g) was washed with ultrapure distilled water several times before being dried at 65 °C for 24 h. The dried An sample was ground to less than 100 μm and stored in a desiccator for further use.

About 10 g of the An powder was homogeneously mixed with 0.2 g of MWCNTs (solid/solid ratio of 50/1). This minute quantity of MWCNTs was selected to achieve the planned goal with minimal costs. The prepared mixture was calcinated for 2 h at 950 °C. After being cooled down to room temperature, the calcinated mixture was gently ground and stored for further application under the name of An/CNT composite.

### 2.3. Materials characterization

The XRD patterns of An, MWCNTs, and the An/CNT composite were obtained by a Philips diffractometer (model APD-3720) in the 2*θ* range of 5°–80° with a scanning speed of 5° min^−1^. The functional groups of the An, MWCNTs, An/CNT and the spent An/CNT were studied by FT-IR (FTIR-2000/Bruker) from 400 to 4000 cm^−1^. Whereas, the morphological features of the An, An/CNT and the spent An/CNT were investigated by an SEM (JEOL/JSM-6700F/Tokyo-Japan). The MWCNTs and An/CNT were separately characterized by a TEM (JEOL Model 2010/Japan) to confirm the multi-walled nature of these tubes and the success of their impregnation process within the flakes of the pristine An, respectively.

The surface area, pore volume and pore size of both raw An and the An/CNT composite were determined by a surface area analyzer (Tristar II analyzer) after degassing at 200 °C for 1 h. The Brunauer–Emmet–Teller^17^ and Barrett–Joyner–Halenda^18^ equations were used to estimate the BET surface area (*S*_BET_), pore volume and pore size of both An and An/CNT, respectively.

### 2.4. Batch studies

For the isotherm study, 30 mg of the An/CNT composite and 100 mL of MO solution with initial concentrations of 40, 60, 80, 100, 120, 140 mg L^−1^ were put into 250 mL centrifuge bottles and mixed on an orbital shaker (SHO-2D/Germany) for 2 h at 200 rpm. The mixtures were then centrifuged (Mikro 120/Hettich/UK) for 15 min at 10 000 rpm to isolate liquid from solid. The residual MO concentration in the supernatant was determined by a UV-visible spectrophotometer (Shimadzu/Model UV 1601/Japan) at *λ*_max_ = 468 nm. Eqn (1) given in Table 1 was used to determine the sorbed amount of MO by the An/CNT composite at equilibrium (*q*_e_, mg g^−1^).

**Table 1:** Equilibrium and kinetic equations that express the MO sorption by An/CNT composite

Equation no.	Linear form	Parameters
Eqn (1)	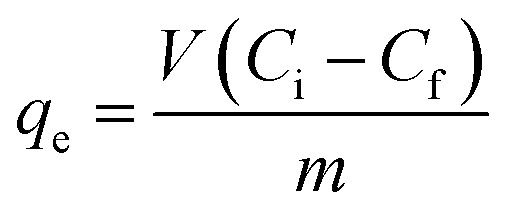	*q*_e_ (mg g^−1^): sorbed amount of MO at equilibrium
		*C*_i_: the initial MO concentration in solution (mg L^−1^)
		*C*_f_: the concentration of MO at equilibrium (mg L^−1^)
		*V*: the volume of MO solution (mL)
		*m*: the mass of An/CNT composite (mg)
Eqn (2)	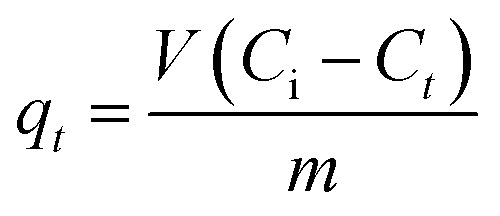	*q*_*t*_ (mg g^−1^): sorbed amount of MO at time *t*
		*C*_i_: the initial MO concentration in solution (mg L^−1^)
		*C*_*t*_: the concentration of MO (mg L^−1^) at time *t*
		*V*: the volume of MO solution (mL)
		*m*: the mass of An/CNT composite (mg)
Eqn (3)	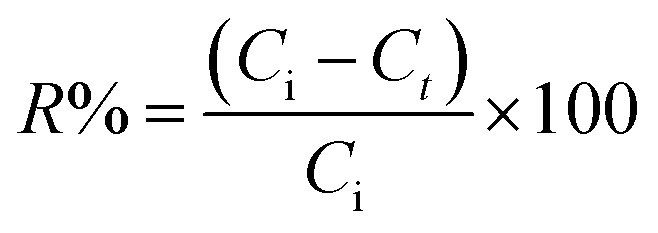	*R*%: removal efficiency of MO by An/CNT
		*C*_i_: the initial MO concentration in solution (mg L^−1^)
		*C*_*t*_: the concentration of MO (mg L^−1^) at time *t*

For the kinetic experiments, all above experimental conditions were followed except that the initial MO concentration was fixed at 100 mg L^−1^ and the pH was adjusted to 3.0. The mixtures were then shaken for 5–240 min. The sorbed amount (*q*_*t*_, mg g^−1^) and the MO removal efficiency (*R*%) by the An/CNT composite were estimated using eqn (2) and (3), respectively (Table 1).

All tests were conducted in triplicate at ambient temperature and the average values were used. For quality assurance/quality control (QA/QC), the input MO concentrations in all tests were measured separately and the percentage of error was within 3%.

### 2.5. Effect of pH, sorbent dose, concentration, agitation time and speed

To evaluate the impact of the applied parameters on the MO sorption by the An/CNT composite, the experiments were performed according to the prevailing conditions given in Table 2.

**Table 2:** The applied experimental parameters and the prevailing conditions during the conduction of the MO adsorption experiments by An/CNT composite

Investigated parameter	Conditions								The other parameters
pH	2	3^a^	4	5	7	8	9	10	100 mg L^−1^ of MO initial conc., 30 mg dose, 200 rpm/2 h (agitation time/speed)
Dose (mg)	10	20	30^a^	40	60	80	100		pH (3.0), 200 mg L^−1^ of MO initial conc., 200 rpm, 2 h (agitation time/speed)
Agitation time (min)	5	15	30	60	120^a^	240			pH (3.0), 100 mg L^−1^ of MO initial conc., 30 mg dose, 200 rpm agitation speed
MO initial conc., (mg L^−1^)	40	60	80	100^a^	120	140			pH (3.0), 30 mg dose, 200 rpm, 2 h (agitation time/speed)
Agitation speed (rpm)	50	100	150	200^a^	250	300			pH (3.0), 100 mg L^−1^ of MO initial conc., 30 mg dose, 2 h of agitation

a Optimum conditions.

## 3. Results and discussion

### 3.1. Characterization of An, MWCNTs, and An/CNT composite

**Fig. 1:**
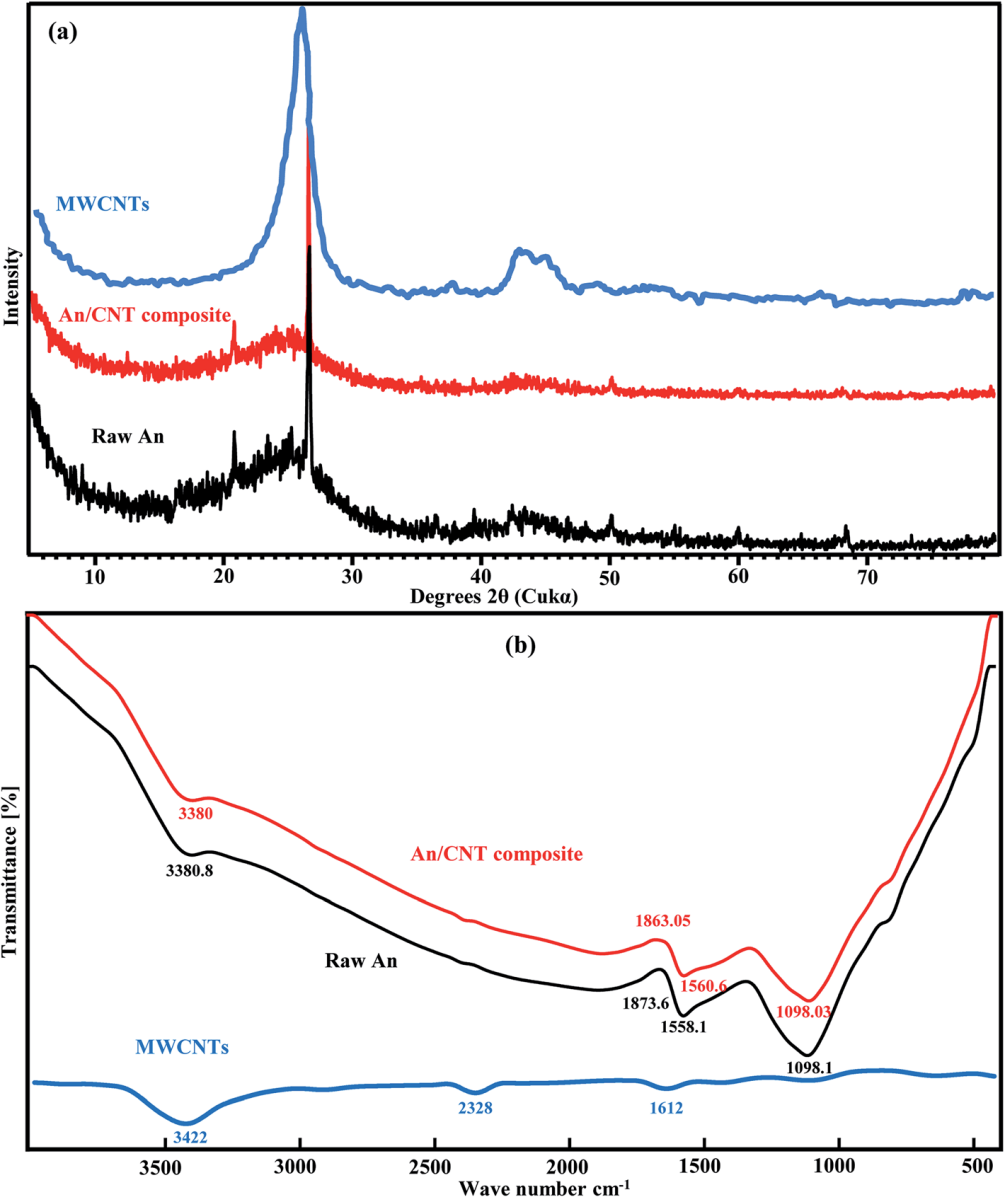
XRD pattern of An, MWCNTs, and An/CNT composite (a), FT-IR spectra of An, MWCNTs and An/CNT composite (b).

The XRD pattern of the raw An displayed a big hump at 2*θ* ≈ 20–30°, suggesting its amorphous nature. The sharp peak at 26.6° is related to the (002) reflection plane of graphite with *d*-spacing 3.348 Å,^19^ meanwhile, the minor peak at 2*θ* = 20.84° could be attributed to γ-phase carbon.^19^ Moreover, the minor peaks at 2*θ* ≈ 50.12, 59.95 and 68.37° are from a minute amount of quartz (Fig. 1a).

In contract, the XRD pattern of the MWCNTs displayed a wide and strong diffraction peak attributed to the (002) reflection of graphite (Fig. 1a). The width at half height is about 3°, reflecting the nanometer nature of the MWCNTs. Using the Scherrer equation, the calculated average grain size is about 3 nm. Moreover, the MWCNTs pattern also displayed broad peaks at 2*θ* 43–46°, which could be assigned to the (101) reflection of graphite. Unlike the An results, the MWCNTs pattern displayed no quartz peaks, confirming their high degree of purity and their low organic carbon content (<3%). Despite the impregnation of MWCNTs to An by calcination, the produced An/CNT composite maintained the inherited diffraction peaks of its An precursor, with no noticeable change in its pattern, owing to the large An/CNT ratio of 50/1 (Fig. 1a). But with close and deep view to the XRD parameters of the (002) reflection plane of graphite in An before and after its impregnation with CNTs (Table 3), we can notice an increase in the *d*_*c*_ (height between the successive layers along *c* direction), *L*_*c*_ (height of crystallite stacking), *L*_a_ (size of crystallite) and *n* (no. of stacking layers, *L*_*c*_/*d*_(002)_) in the An/CNT composite. This increase in some geometrical parameters of An/CNT confirms the success of the CNTs impregnation process within the layers and flakes of the pristine An. It also indicate the critical role played by the impregnated CNTs not only in the promotion of the graphitization degree of An by making its microcrystalline structure more orderly arranged and more densely packed,^20^ but also in raising the oxidation temperature of An and in delaying its complete consumption at such extreme calcination conditions (*i.e.* the impregnation increased the melting point of An). This later deduction was affirmed by non-presented calcination experiment (calcination at 950 °C/2 h) conducted for the pristine An without the addition of CNTs. The LOI after this experiment was 90%, while greyish white ash remains were 10%.

**Table 3:** XRD parameters of An before and after impregnation with CNTs by calcination at 950 °C/2 h

Sample	2*θ*_002_ (°)	*d* spacing (height between adjacent layers in the *c* direction), *d*_*c*_ (Å)	Crystallite stacking height *L*_*c*_ (Å)	Crystallite size *L*_a_ (Å)	Staking layer number *n* (*L*_*c*_/*d*_002_)
An	26.63	3.348	270.59	404.76	80.82
An/CNT composite	26.58	3.353	355.70	639.53	106.1

The FT-IR investigations revealed a close and logical match in the spectra of the raw An and its derivative An/CNT composite (Fig. 1b), again owing to the An/CNT ratio of 50/1. The functional groups matched the frequency and intensity of OH stretching group (silanol) at 3380 cm^−1^ (ref. 21 and 22) and the stretching mode of Si–O asymmetric group at 1098 cm^−1^ due to the presence of minute quartz.^23,24^ The carboxylic group shifted from 1873.7 to 1863.05 cm^−1^ (ref. 25) and the C–C, C=N or C–H aromatic bending group moved slightly from 1558.1 to 1560.6 cm^−1^.^21,26^ Moreover, the FT-IR spectra of the applied MWCNTs in the impregnation process revealed that OH (3422 cm^−1^) and C=O (1612 cm^−1^) are the main characterizing functional groups^11^ (Fig. 1b). These groups are slightly shifted to lower frequencies in the produced An/CNT composite as a sign for the success of the impregnation process.

**Fig. 2:**
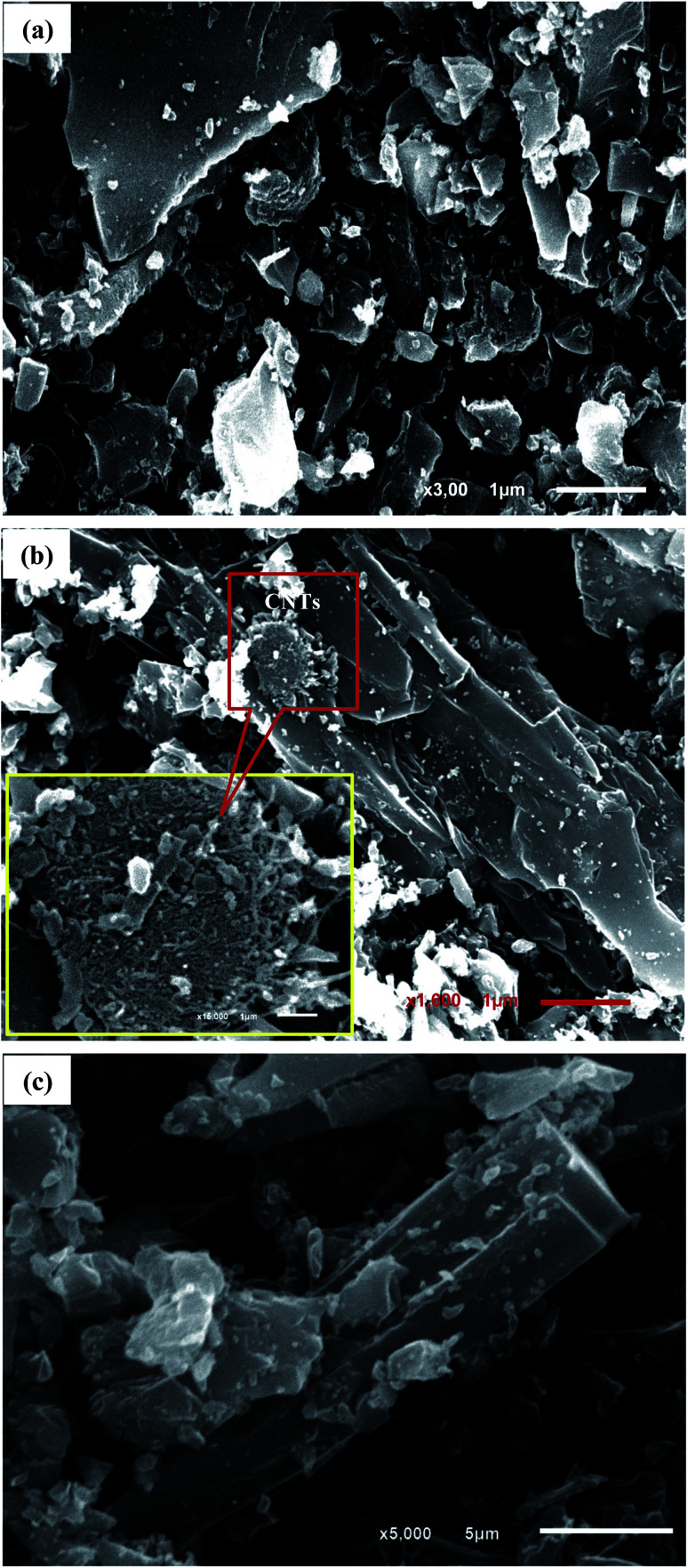
SEM images showing: the pristine An with flaky particles and various grain sizes (a), An/CNT composite with smoother surface and MWCNTs impregnation (b), An/CNT composite with well-developed hexagonal crystals of graphite (c).

The SEM images confirmed the porous and flaky nature of raw An with different grain sizes and shapes (Fig. 2a). On the other hand, the impregnation of MWCNTs by calcination to produce An/CNT composite (Fig. 2b) contributed to the improvement of the degree of graphitization of the pristine An through the appearance of well-developed hexagonal crystals of graphite (Fig. 2c).

**Fig. 3:**
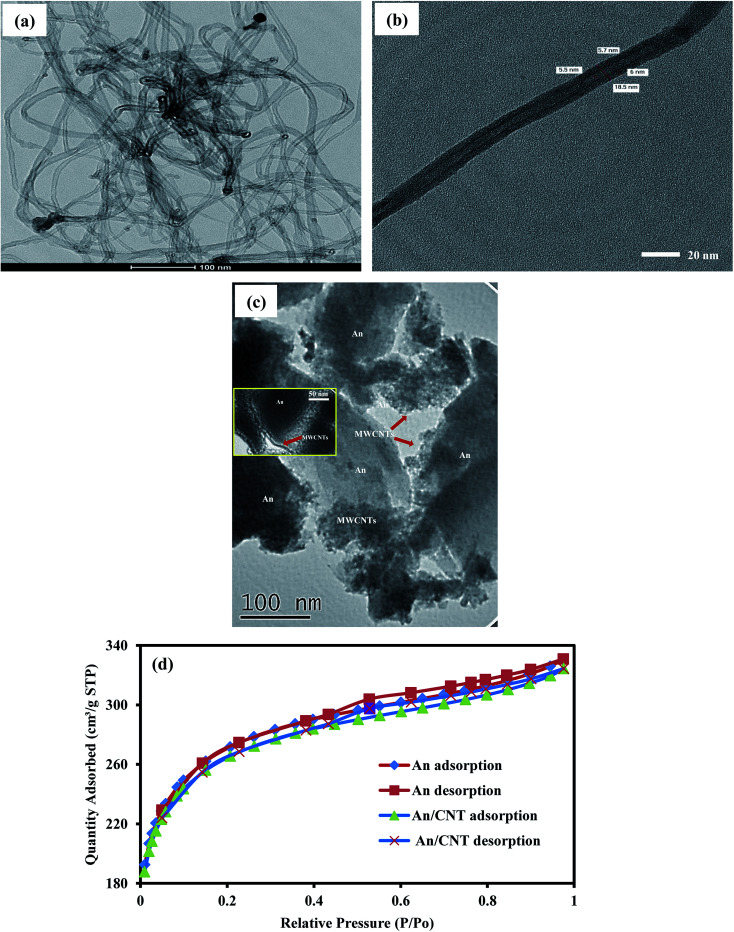
TEM images of the employed MWCNTs in the production of An/CNT composite with nest structure, multi-walled nature and high aspect ratio (a and b), TEM image of An/CNT showing the success of the CNT impregnation process (c), nitrogen adsorption–desorption isotherms of An and An/CNT composite (d).

The TEM images of the MWCNTs confirmed their nest structure and multi-walled nature (Fig. 3a and b). The dimensional analyses of these tubes revealed a high aspect ratio with outer diameters range from 18–23 nm and inner ones from 2–6 nm and up to 10 μm length, confirming the particle size as calculated from the XRD pattern. Also the success of the impregnation process of MWCNTs within the flakes of the raw An to prepare An/MWCNTs composite is confirmed using the TEM imaging technique (Fig. 3c with inset).

The nitrogen adsorption/desorption isotherm revealed that An and An/CNT composite are of type I isotherm of the IUPAC classification with H_4_ hysteresis loops (Fig. 3d). These results designate both An and An/CNT as microporous materials.^21,27^ This is in consistency with the micropore volume (*V*_micro_) representing 59% and 83% of the total pore (*V*_t_) in the An and An/CNT, respectively. Also, the geometrical parameters (Table 4) revealed that impregnation of MWCNTs into An by calcination resulted in a slight reduction in *S*_BET_, *V*_t_, and pore diameter (*D*_p_) of the prepared An/CNT composite. This reduction could be related to the condensed packing of the An/CNT particles (*i.e.* pore destruction) and the blocking of the An porous structure with the impregnated CNTs that hindered the passage of nitrogen.^28^ In spite of this reduction, the geometrical parameters of the An/CNT at still very high with *S*_BET_ of 870.1 m^2^ g^−1^ and *V*_t_ of 0.264 cm^3^ g^−1^.

**Table 4:** Textural parameters of An and An/CNT composite obtained from the nitrogen adsorption isotherms

Sample	Surface area (m^2^ g^−1^)			Pore volume (cm^3^ g^−1^)			Average pore diameter (nm)
	BET (*S*_BET_)	Mesopores (*S*_meso_)	Micropores (*S*_micro_)	Total pore volume (*V*_t_)	Mesopore volume (*V*_meso_)	Micropore volume (*V*_micro_)	*D*_p_
An	891	438.9	452.1	0.38	0.155	0.225	2.49
An/CNT	870.1	430.74	439.34	0.264	0.045	0.219	1.34

### 3.2. Effect of solution pH

**Fig. 4:**
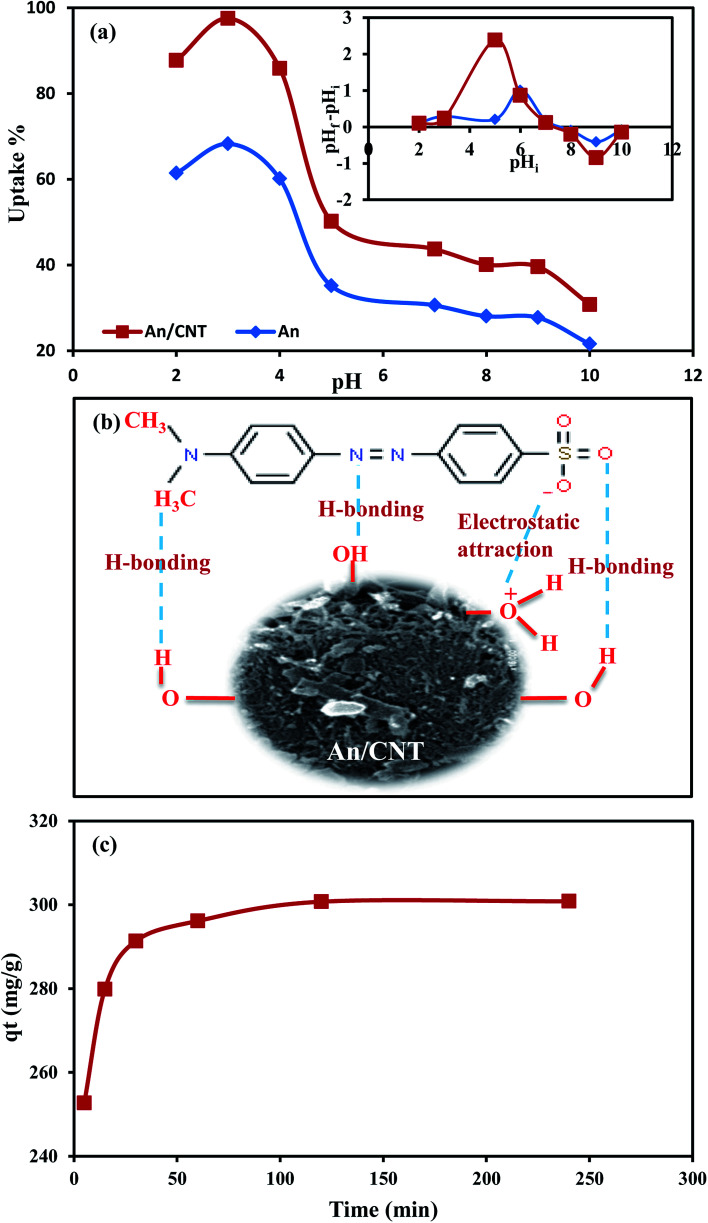
Effect of pH on MO uptake by An and An/CNT composite with their point of zero charge as inset (a), schematic diagram showing the mechanisms of MO sorption by An/CNT composite (b), effect of contact time on MO uptake by An/CNT composite (c).

The MO removal by the An/CNT composite and its precursor An is a pH-dependent process. At pH 2.0–4.0, the MO removal efficiency (*R*%) was very high for both sorbents (Fig. 4a), but the maximum sorption was achieved only at pH 3.0 (*R*% = 68.3 and 97.6% for An and An/CNT composite, respectively), in an agreement with a previous study.^29^ The superior sorption behavior of An/CNT for MO over that of its precursor An, despite the slight decrease in its geometrical parameters, could be related to its higher degree of graphitization as discussed above. Beyond pH 4.0, MO sorption was noticeably declined, especially under basic conditions. This coincides with the point of zero charge (pH_PZC_) determined according to the reported protocol.^30^ These results displayed that the surface of the An/CNT composite and it's An precursor was negative above pH 7.3 and 7.8, respectively (inset in Fig. 4a).

The higher degree of ionization of MO (p*K*_a_ = 3.46)^31,32^ and the protonation of the An and An/CNT surfaces^33^ at pH 3.0, promoted the electrostatic interactions between the binding sites of the studied sorbents (protonated hydroxyl groups) and the negative sulfonate group (–SO_3_^−^) of MO ions in solution. In the protonation process of the An and An/CNT composite surfaces, hydrogen ions act as bridging ligands between the MO anions and the surface of the studied sorbents.

Conversely, the prevalence of the competitive OH^−^ ions in the solution beyond the pH_PZC_ of the sorbents resulted in de-protonation of their surfaces and triggered repulsive forces against MO anions,^34^ leading to a reduction in MO uptake by both sorbents. So, pH 3.0 was considered for conducting the experiments of the succeeding parameters.

Electrostatic interaction cannot be regarded as the only driving force for MO sorption by both An and An/CNT and considerable contributions from other hydrophobic forces (hydrogen bonding) may be counted (Fig. 4b), especially at pH > pH_PZC_.^35,36^ Such forces can be correlated to the hydrophobicity of graphite and organic matter constituents of the addressed sorbents.^37,38^ The hydrogen bonding interaction can be categorized into two styles: (1) Yoshida H-bonding and (2) dipole–dipole H-bonding.^35,36^ The former interaction style occurs between the aromatic rings of the MO and the hydroxyl groups upon addressed sorbents surfaces.^35,36^ Whereas, the latter interaction links the nitrogen and oxygen atoms of the MO (H-acceptor) with the accessible hydrogen atoms of the An and An/CNT hydroxyl and carboxyl groups (H-donor).^35,36,39^ The involvement of H-bonding mechanism was affirmed by the noticeable sorption efficiency of MO by An and An/CNT at pH 8.0–10.0 (21.56 to 30.59% and 30.8 to 43.7%, respectively) regardless of the OH^−^ presence as a competing ions in the solution.

### 3.3. Effect of contact time

At 5 to 30 min agitation time interval, the MO removal was very rapid (from 252.7 to 291.3 mg g^−1^) (Fig. 4c). This sorption behavior was related to the great accessible number of unoccupied active sites for MO uptake on the composite's surface.^33^ Whereas, at mixing time > 30 min and ≤120 min (296.1 and 300.7 mg g^−1^ for 60 and 120 min, respectively), the MO sorption was slow owing to the decrease in the available vacant binding sites on the An/CNT surface. Beyond 120 min, there is no further perceptible increase in the MO adsorption by the sorbent (*q*_*t*_ = 300.8 mg g^−1^ at 240 min). This indicates that equilibrium was attained at 120 min. Therefore, 120 min was deployed as an equilibrium time for the other experiments.

### 3.4. Effect of initial concentration

**Fig. 5:**
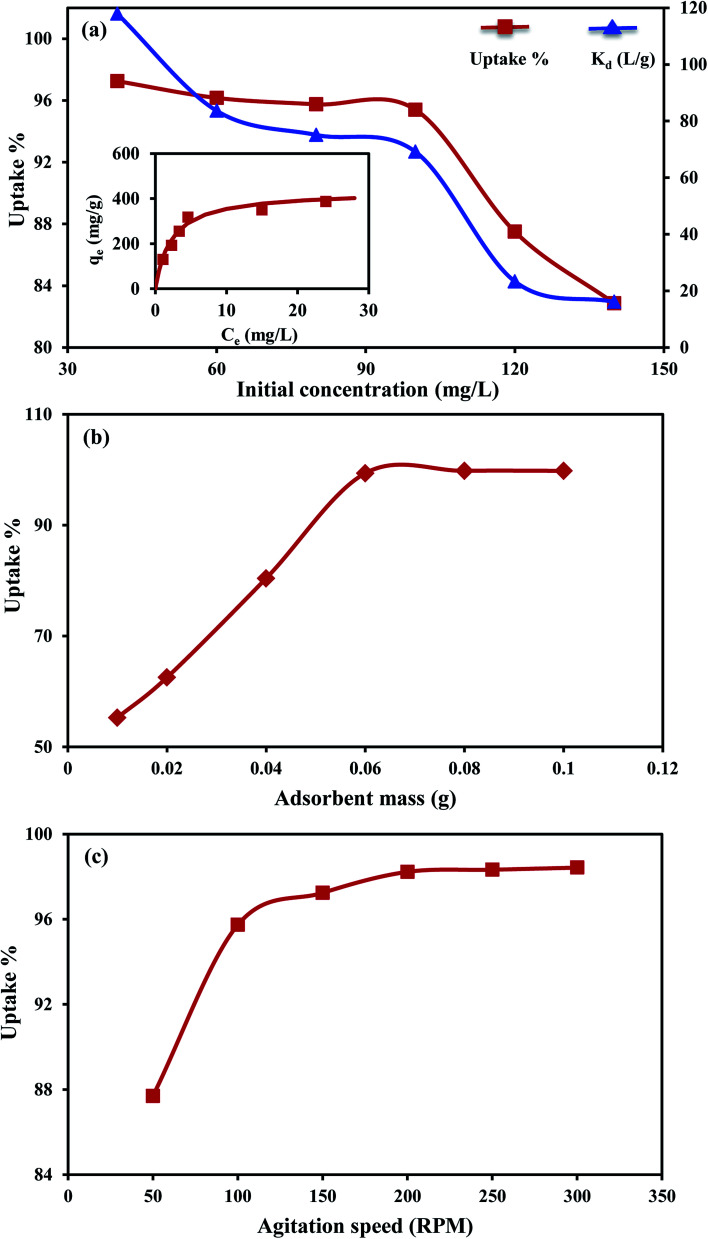
Effect of initial concentration on MO uptake by An/CNT composite and *K*_d_ by An/CNT composite with the inset being the Langmuir fit to the observed data (a), effect of absorbent mass on MO uptake by An/CNT composite (b), effect of agitation speed on MO uptake by An/CNT composite (c).

Increases in initial MO concentration decreased its *R*% from 97.4% to reach 81.9% (Fig. 5a). However, the amount of MO sorbed (*q*_e_, mg g^−1^) increased with raising the initial concentrations. The progressive collision of MO ions with the active sites of the composite with increasing the initial concentration was the key factor in such enhancement. Also, the enhancement of MO sorption was derived by the increase in the mass gradient that acted as a driving force for transferring the MO ions onto the An/CNT surface.^40^ The overall reduction in *R*% at high initial concentrations was associated with the deficiency in the vacant sites that could accommodate the MO ions from solution.^41^

Unlike removal efficiency and sorption capacity that are controlled by the operating conditions, distribution coefficient *K*_d_ can be used as a true metric to merit the adsorbent true performance.^42–44^*K*_d_ is a representative ratio for the analyte concentration among both solid and liquid phases at equilibrium. In the current study, the MO distribution coefficient *K*_d_ revealed a marked decrease from 117.9 to 16.1 L g^−1^ with increasing initial MO concentrations from 40 to 140 mg L^−1^ (Table 5).

**Table 5:** Changes in residual MO concentration, amount of MO sorbed, removal rate (%) and distribution coefficient (*K*_d_) as a function of initial MO concentrations

Dose (mg)	Initial MO conc. (mg L^−1^)	Residual MO conc. (mg L^−1^)	MO sorbed (mg g^−1^)	Removal rate (%)	*K*_d_ (L g^−1^)
30	40	1.1	129.7	97.3	117.9
30	60	2.3	192.3	96.1	83.6
30	80	3.4	255.3	95.7	75.1
30	100	4.6	318	94.9	69.1
30	120	15	350	87.4	23.3
30	140	24	386.7	81.9	16.1

### 3.5. Effect of adsorbent mass

Increasing the composite mass from 0.01 to 0.06 g was accompanied by an increase in MO uptake from 55.3 to 99.4% (Fig. 5b). This justifiable increase in MO uptake was interrelated to the surface area of the adsorbent and hence the accessible sites for MO sorption.^33,45^ Beyond 0.06 g of composite mass, no appreciable increase in MO uptake was observed (≈99.8% for 0.08 and 0.1 g). This indicates that the equilibrium state was attained.

### 3.6. Effect of mixing speed

The sorption of MO under different mixing speeds (50 to 300 rpm) was carefully examined. At the speed from 50–100 rpm, the *R*% rapidly increased from 87.7 to 95.7% (Fig. 5c). But at the speeds >100 to ≤200 rpm, the *R*% increased slowly from 97.2 to 98.3%. Beyond 200 rpm, there is no perceptible increase in *R*%. This indicates that equilibrium was attained at a mixing speed of 200 rpm that was used for all the experiments. In comparison, as the mixing speed increased from 300 to 700 rpm, MO sorption on MWCNTs increased from 25 to 45 mg g^−1^, corresponding to 50–100% *R*% value.^13^

### 3.7. Sorption kinetics

The kinetic data of MO removal by An/CNT composite were fitted to the pseudo-1^st^ order “PFO”, pseudo-2^nd^ order “PSO”, and inter-particle diffusion models. The equations that express the linear formula of these models and the values of their related constants were compiled in Tables 6 and 7.

**Table 6:** Sorption kinetics models for MO uptake by An/CNT composite

Kinetic model	Linear form	Parameters	Ref.
Pseudo-first order	ln(*q*_e_ − *q*_*t*_) = ln *q*_e_ − *k*_1_*t*	*q*_*t*_ (mg g^−1^): removed amount of MO at time *t*	53
		*q*_e_ (mg g^−1^): equilibrium sorption uptake	
		*K*_1_ (g mg^−1^ min^−1^): rate constant of the first-order adsorption	
		*k*_1_: −slope	
		*q*_e_ (cal): EXP^(intercept)^	
Pseudo-second order	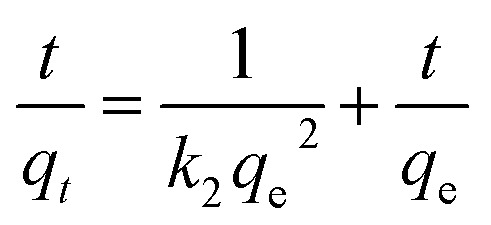	*q*_*t*_ (mg g^−1^): removed amount of MO at time *t*	54
		*q*_e_ (mg g^−1^): equilibrium sorption uptake	
		*K*_2_ (g mg^−1^ min^−1^): rate constant of the second-order adsorption	
		*q*_e_ (cal) = 1/slope	
		*k*_2_ = (slope) × 2/intercept	
Intra-particle diffusion	*q*_*t*_ = *k*_p_*t*^1/2^ + *C*	*q*_*t*_ (mg g^−1^): removed amount of MO at time *t*	55
		*K*_p_ (mg g^−1^ min^−0.5^): intra-particle diffusion rate constant	
		*C* (mg g^−1^): intercept of the line which reflects the thickness of the boundary layer	
		*k*_p_ = slope	
		*C* = intercept	

**Table 7:** The kinetic parameters of the MO sorption by An/CNT composite

Initial MO concentration (mg L^−1^)	*q* ^exp^_e_ (mg g^−1^)	Kinetic model								
		Pseudo-first order			Pseudo-second order			Intra-particle diffusion		
		Parameters			Parameters			Parameters		
		*K*_1_ (min^−1^)	*q* ^cal^_e_ (mg g^−1^)	*R* ^2^	*K*_2_ (g mg^−1^ min^−1^)	*q* ^cal^_e_ (mg g^−1^)	*R* ^2^	*K*_p_ (mg g^−1^ min^−0.5^)	*C* (mg g^−1^)	*R* ^2^
100	300.1	0.0405	43.98	0.9219	0.00303	303.0	1	4.53	262.8	0.9921

**Fig. 6:**
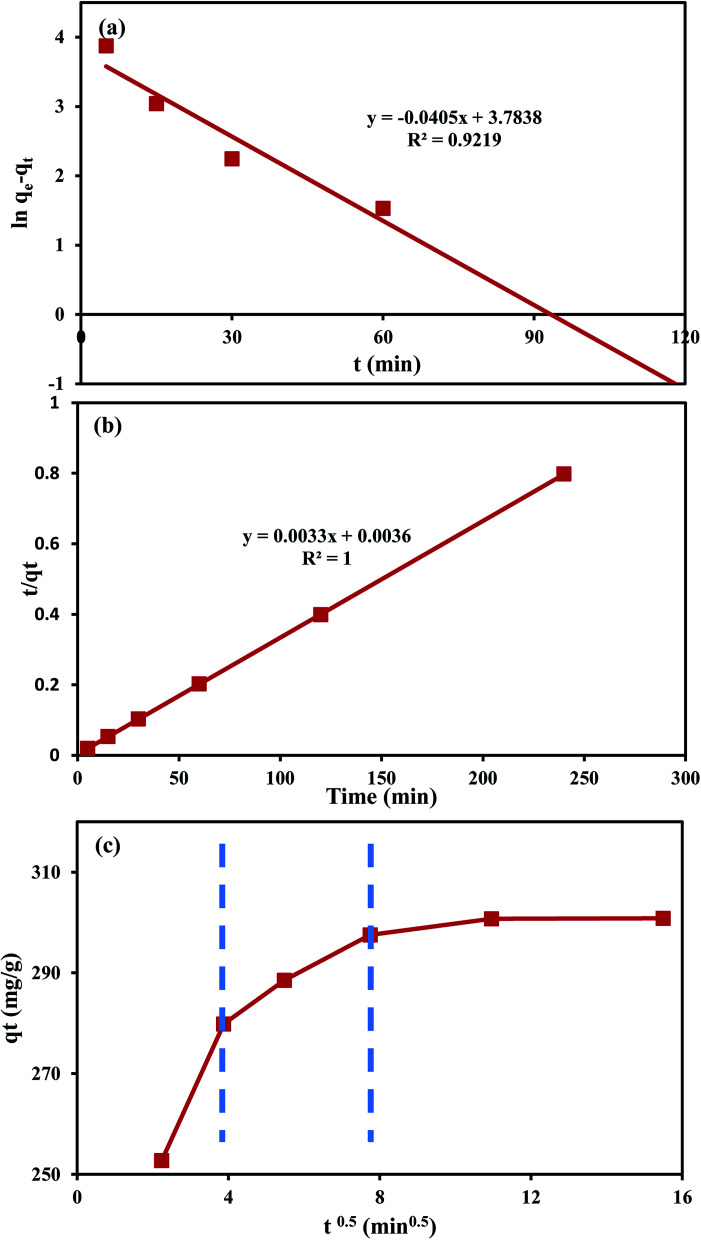
Plot of first-order kinetic model for MO uptake by An/CNT composite (a), plot of second-order kinetic model for MO uptake by An/CNT composite (b), intra-particle diffusion model for MO uptake by An/CNT composite (c).

The fitted results using the PSO equation are better than those using the PFO one (Fig. 6a and b), as expressed by the high determination coefficient (*R*^2^ = 1) of the PSO equation compared to that of the PFO (Table 7) with an experimental (*q*^exp^_e_) and theoretical (*q*^cal^_e_) values of 300.1 and 303.0 mg g^−1^, respectively. The PSO rate constant is 3 × 10^−3^ g mg^−1^ min^−1^ for MO sorption on An/CNT in comparison to 1–6 × 10^−3^ g mg^−1^ min^−1^ for MO sorption on MWCNTs only.^13^ Similarly, the PFO rate constant is 0.04 min^−1^, in comparison to 0.02–0.03 min^−1^ for MO sorption on MWCNTs.^13^ These results suggested synergistic effects of An with MWCNTs in comparison to MWCNTs alone.

On the other hand, the graphical presentation of the intra-particle diffusion model (*q*_*t*_*vs. t*^0.5^) for MO sorption by An/CNT composite yielded a multi-linear plot deviating out of the origin (Fig. 6c). This behavior affirmed that intra-particle diffusion was not the only rate-principal step in MO adsorption process.^46^ Meanwhile, the boundary diffusion layer thickness of (*C* = 262.82 mg g^−1^) reflected the effective involvement of the composite's surface in MO removal, in good agreement with the *S*_BET_ results (Table 4).

Such a three-step graph designated the intervention of various diffusion styles in the MO removal process.^31,47^ The 1^st^ step implied the quick transfer of MO anion to the composite's exterior surface *via* a hydrodynamic boundary layer “film or external diffusion”.^25,36^ The 2^nd^ step indicated the MO slow diffusion from the external surface of the composite into its pores and/or pore-wall interstices, indicating the domination of the intra-particle diffusion.^48^ Therefore, the *K*_p_ and *C* values of the intra-particle diffusion equation were calculated from this step (Table 7). Whereas, the 3^rd^ plateau step referred to the equilibrium state as indicated by the nearby fixed rate of MO sorption by the An/CNT composite whatever the time progress.

### 3.8. Sorption isotherms

**Fig. 7:**
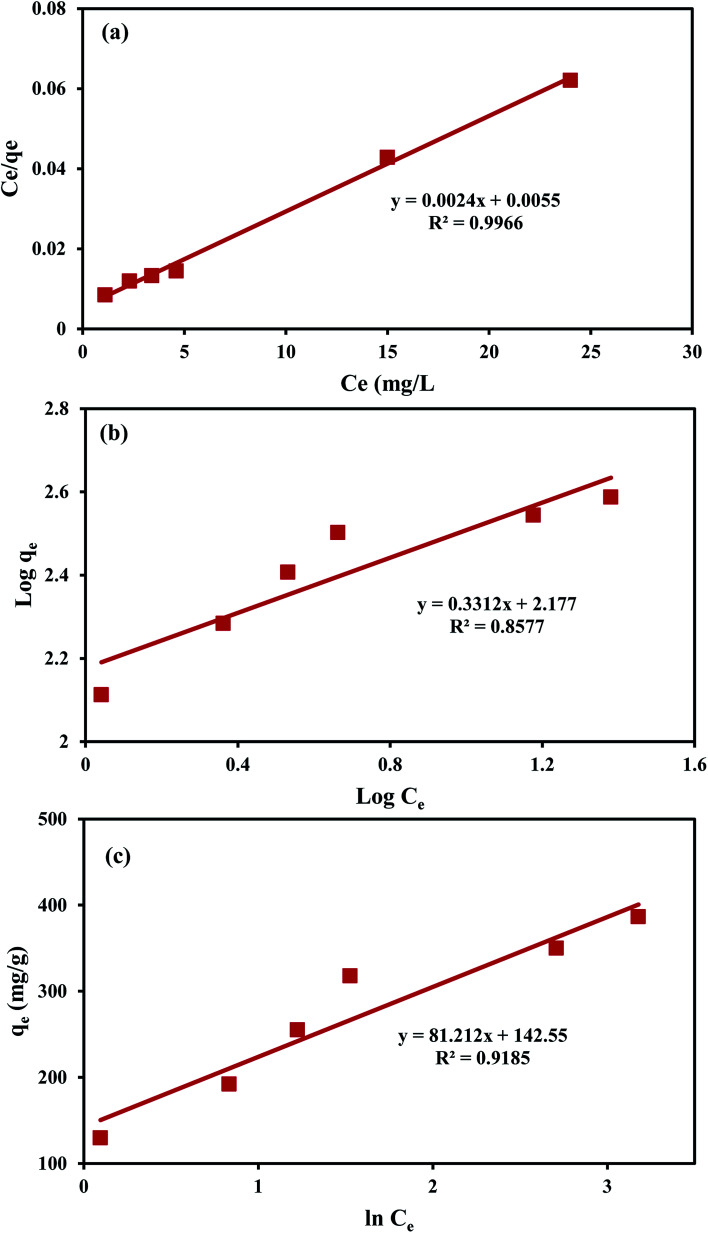
Langmuir isotherm for MO uptake by An/CNT composite (a), Freundlich isotherm for MO uptake by An/CNT composite (b), Temkin isotherm for MO uptake by An/CNT composite (c).

The linear forms of Langmuir, Freundlich, and Temkin models were used to elucidate the interactions between MO anions and the active sites on the An/CNT composite's surface (Table 8). The parameters of these equations were calculated using *C*_e_*vs. C*_e_/*q*_e_, log *C*_e_*vs.* log *q*_e_ and ln *C*_e_*vs. q*_e_ plots, respectively (Fig. 7a–c, Table 9).

**Table 8:** Sorption isotherm models for MO uptake by An/CNT composite

Isotherm Model	Linear form	Parameters	Ref.
Langmuir	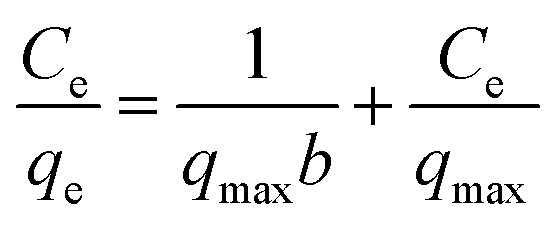 , *R*_L_ = 1/(1 + *bC*_0_), *R*_L_ > 1 (unfavorable adsorption), *R*_L_ = 1 (linear adsorption), 0 < *R*_L_ < 1 (favorable adsorption), *R*_L_ = 0 (irreversible adsorption)	*C*_e_ (mg L^−1^): equilibrium concentration of the residual MO in the solution	56 and 57
		*q*_e_ (mg g^−1^): removed amount of MO at equilibrium	
		*q*_max_ (mg g^−1^): maximum adsorption capacity	
		*b* (L mg^−1^): Langmuir constant	
		*q*_max_ = 1/slope	
		*b* = slope/intercept	
		*C*_0_: initial MO concentration	
		*R*_L_: equilibrium parameter of Langmuir equation	
Freundlich	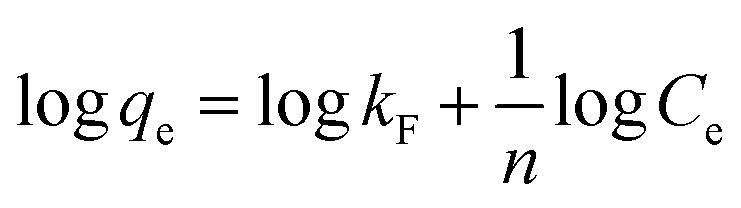	*C*_e_ (mg L^−1^): equilibrium concentration of the residual MO in the solution	58
		*q*_e_ (mg g^−1^): removed amount of MO at equilibrium	
		*K*_F_ (mg g^−1^): MO adsorption capacity	
		*n*: heterogeneity factor	
		*k*_F_ = 10^intercept^	
		1/*n* = slope	
Temkin	qe = B ln A + B ln Ce, B = RT/b	*A* (L g^−1^): Temkin isotherm constant (the equilibrium binding constant corresponding to the maximum binding energy)	59
		*B* (J mol^−1^): Temkin constant related to heat of sorption	
		*b*: Temkin isotherm constant	
		*R*: the gas constant (8.314 J mol^−1^ K^−1^)	
		*T*: the absolute temperature at 298 K	
		*A* = EXP^(intercept/slope)^	
		*b* = slope	

**Table 9:** The isotherm parameters of the MO adsorption by An/CNT composite

Initial MO concentration (mg L^−1^)	Isotherm model parameters									
	Langmuir				Freundlich			Temkin		
	*q*_max_ (mg g^−1^)	*b* (L mg^−1^)	*R* ^2^	*R*_L_	1/*n*	*K*_F_ (mg g^−1^)	*R* ^2^	*B* (J mol^−1^)	*A* (L g^−1^)	*R* ^2^
40–140	416.7	0.436	0.997	0.016–0.054	0.331	150.31	0.858	30.51	5.79	0.919

In view of the outcomes of the isotherm studies, the fitting of the MO sorption data by the applied equations followed: Langmuir > Temkin > Freundlich based on their *R*^2^ values (0.997, 0.919 and 0.858, respectively), suggesting that the MO uptake by the studied composite was homogeneous “mono-layer” in nature through iso-energetic binding sites on the An/CNT composite's surface.^41,49^ Furthermore, the *R*_L_ (Langmuir equilibrium parameter) values (0.016–0.054) that was calculated using “*R*_L_ = 1/(1 + *bC*_0_)” equation (Tables 8 and 9), confirmed a favorable sorption of MO ions (favorable, 0 < *R*_L_ < 1) by the composite and the appropriateness of Langmuir model to describe the sorption data well with remarkably high *q*_max_ (416.7 mg g^−1^).

A comparison of the *q*_max_ and PC (partition coefficient) of MO uptake onto An/CNT composite with other sorbents revealed the superiority of the composite over the other investigated natural, modified, and synthetic sorbents (Table 10). It can be seen that the An/CNT composite in terms of *q*_max_ and PC (416.7 mg g^−1^ and 197.5 mg g^−1^ μM^−1^, respectively) is more efficient than the different cited types of clay minerals, bottom ash, de-oiled soya, nanoparticles, modified layered double hydroxides, pure MWCNTs, functionalized MWCNTs, activated carbons and synthetic nano-composites, except for MWCNTs/Fe_3_O_4_/PANI magnetic composite on the level of *q*_max_ only (Table 10). Therefore, with such metrics, the An/CNT composite can be nominated as a superior, economical and efficient adsorbent for MO removal on industrial scale.

**Table 10:** Sorption capacity (*q*_max_) and partition coefficient (PC) for MO by some natural, modified and synthetic materials in comparison with the An/CNT composite of the present study: *q*_max_ obtained from the Langmuir constant, PC = *q*_max_/residual MO concentration

Adsorbent	Initial MO concentration (mg L^−1^)	Residual MO concentration (μM)	Adsorption capacity *q*_max_ (mg g^−1^)	pH/*T* (K)	Partition coefficient (mg g^−1^ μM^−1^)	Reference
Bottom ash	32.7	1.4^b^	3.62	3.0/333	2.6^b^	60
De-oiled soya	32.7	0.2^b^	16.66	3.0/333	83.3^b^	60
Activated clay	80	—	16.78	7.0/313	—	61
Maghemite/chitosan nano-composite films	20	—	29.41	3.0/330	—	62
Bentonite	—	—	33.8	4.5/298	—	63
Organic matter rich clays (OMRC)	100	10.4	41.67	2.0/298	4.01	41
Calcinated organic matter rich clays (COMRC)	100	39.6	34.48	2.0/298	087	41
Functionalized-CNTs with 3-aminopropyltriethoxysilane loaded TiO_2_ nanocomposites	5	0.92^a^	42.85	6.5/298	46.52^a^	39
Multi-walled carbon nanotubes	—	—	52.86	2.3/333	—	8
Activated carbon (AC)	10	116.12^a^	78.7	4.5/300	0.48^a^	11
Multi-walled carbon nanotubes (MWCNTs)	10	95.3^a^	106.3	4.5/300	0.99^a^	11
Multi-walled carbon nanotubes/activated carbon (MWCNTs/AC)	10	35.99^a^	196.1	4.5/300	5.31^a^	11
FeOOH/carbonized bacterial cellulose (FeOOH/CBC) nanocomposite	50	5.8^b^	107.7	6.0/333	18.6^b^	64
Zn–Al layered double hydroxides intercalated with surfactant (DS-Zn/Al LDHs)	30	1.8^b^	114.9	7.0/298	63.8^b^	65
CuMgAl layered double hydroxide (CuMgAl LDH)	20	1.2^b^	123.5	7.0/298	101.2^b^	66
Activated carbon/Fe_3_O_4_ nanoparticle composites (10Fe_3_O_4_/PAC-HNO_3_)	—	—	303.03	5.0/303	—	27
Powdered activated carbon modified by HNO_3_	—	—	384.62	5.0/303	—	27
MWCNTs/Fe_3_O_4_/PANI magnetic composite	150	189.41^a^	446.25	4.5/298	2.36^a^	67
An/CNT composite	100	2.11	416.7	3.0/298	197.5	Current study

a These data were derived from Ahlawat *et al.*, 2020 (ref. 11) (Table 6) with the proper correction for the residual MO concentration.

b These data were derived from Grover *et al.*, 2019 (ref. 65) (Table 3) with the proper correction for the residual MO concentration.

With a 10% of the MO sorption capacity of 416.7 mg g^−1^, the equilibrium MO concentration was only 0.23 mg L^−1^. On the other hand, if breakthrough at 10% of the initial MO concentration is deemed as effective, the An/CNT could treat a MO containing wastewater at an initial concentration of 110 mg L^−1^. Thus, the research from this study suggested extreme effectiveness of An/CNT for the removal of MO.

### 3.9. Characterization of the spent sorbent

**Fig. 8:**
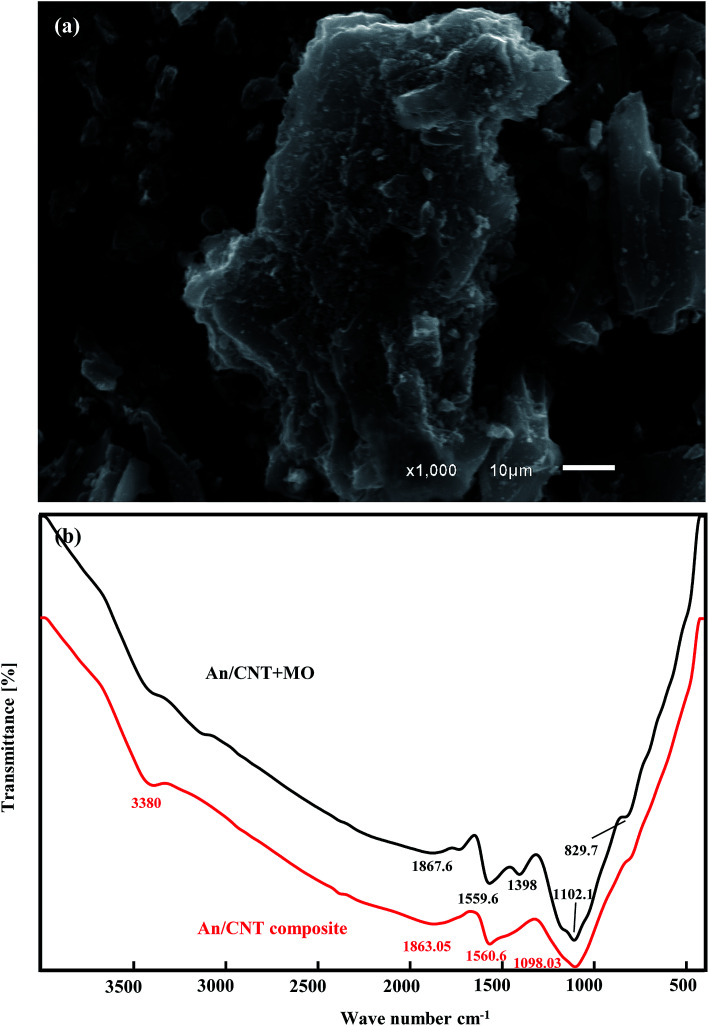
SEM image showing the agglomeration of An/CNT composite after MO uptake (a), FT-IR spectra of An/CNT composite before and after MO uptake (b).

The SEM images of the An/CNT composite after MO sorption revealed that its smooth surface was approximately obliterated due to grain agglomeration by the sorbed MO, resulting in a rough surface with some micro-cavernous features instead (Fig. 8a). This undoubtedly confirms the successful contribution of the binding sites on the composite's surface in the MO adsorption process.

The FT-IR spectrum of the spent composite (Fig. 8b) displayed that the peak that was ascribed to the OH group in the An/CNT spectra was nearly obliterated, while the peaks of the Si–O, C–C (C–H or C=N) and carboxylic groups were shifted to 1102.1, 1559.6 and 1867.6 cm^−1^ frequencies after MO loading. Moreover, the spectrum of the spent composite showed the emergence of a new peak that was related to the symmetric vibration of (CH_3_)_3_ group at 1398 cm^−1^.^50^ Also, the new peak at 3200 cm^−1^ of the NH group may be attributed to the protonation of MO in acidic media. These alterations in the spent composite spectra affirm the involvement of the oxygen-bearing groups on the composite's surface in the MO sorption process.^51,52^

## 4. Conclusion

Through the current study, the following deductions could be formulated:

• The impregnation of MWCNTs into the raw An by calcination at 950 °C for 2 h played a critical role in enhancing its complete transformation into greyish white ash at equivalent calcination condition.

• The produced An/CNT composite approximately maintained the inherited diffraction pattern, FT-IR spectra, and the microporous nature of the An precursor.

• The slight reduction of the geometrical parameters (*S*_BET_, *V*_micro_, *V*_t_, and *D*_p_) of the An/CNT composite, compared to the precursor An, was ascribed mainly to the blocking of the An porous structure with the impregnated CNTs, the dense packing associating An graphitization and organic matters oxidation *via* calcination. All these morphological changes contributed in An/CNT composite with homogenous surface and higher MO removal efficiency.

• The MO isotherm and kinetic data were described well by the Langmuir and PSO models, respectively.

• The homogenous MO sorption by the An/CNT composite was a pH-dependent process and the maximum monolayer coverage (*q*_max_ = 416.7 mg g^−1^) was attained at pH 3.0 and ambient temperature.

• The multi-linearity of intra-particle diffusion fitting unquestionably affirmed that the external (film) (0 < *T* ≤ 15 min) and intra-particle diffusions (15 < *T* ≤ 60 min) were the main driving steps for MO sorption by An/CNT composite.

• Electrostatic interaction was the principal driving mechanism for MO sorption by An/CNT at pH < pH_PZC_, *via* the protonation process of its oxygen-holding groups. While Yoshida and dipole–dipole H bonding mechanisms can explain the MO sorption by the addressed composite, especially at pH > pH_PZC_.

• Finally, the An/CNT composite is an eco-friendly, reliable and affordable sorbent for the remediation of MO contaminated water.

## Conflicts of interest

There are no conflicts of interest to declare.

## Supplementary Material
